# Characterization of a de novo sSMC 17 detected in a girl with developmental delay and dysmorphic features

**DOI:** 10.1186/s13039-017-0312-x

**Published:** 2017-03-23

**Authors:** Lana Stavber, Sara Bertok, Jernej Kovač, Marija Volk, Luca Lovrečić, Tadej Battelino, Tinka Hovnik

**Affiliations:** 1 University Children’s Hospital, University Medical Centre Ljubljana, Unit for Special Laboratory Diagnostic, Vrazov trg 1, SI-1525 Ljubljana, Slovenia; 2 Department of Pediatric Endocrinology, Diabetes and Metabolic Diseases, University Children’s Hospital, UMC, Ljubljana, Slovenia; 3 Clinical Institute of Medical Genetic, UMC, Ljubljana, Slovenia; 4 Faculty of Medicine, University of Ljubljana, Ljubljana, Slovenia

**Keywords:** Small supernumerary marker chromosome, Developmental delay, Speech delay, Dysmorphic features, fluorescence *in situ* hybridization

## Abstract

**Background:**

The majority of small supernumerary marker chromosome cases arise *de novo* and their frequency in newborns is 0.04%. We report on a girl with developmental delay and dysmorphic features with a non-mosaic *de novo* sSMC that originated from the pericentric region of q arm in chromosome 17.

**Case presentation:**

The girl presented with developmental delay, speech delay, myopia, mild muscle hypotonia, hypoplasia of orbicular muscle, poor concentration, and hyperactivity. Main dysmorphic features included: round face, microstomia, small chin, down-slanting palpebral fissures and small lobules of both ears. At present, her developmental abilities are still delayed for her chronological age but she is making evident progress with speech.

A postnatal array comparative genomic hybridization showed a 2.31 Mb genomic gain indicating microduplication derived from pericentric regions q11.1 and q11.2 of chromosome 17. Additional conventional cytogenetic analysis from peripheral blood characterized the karyotype as 47,XX,+mar in a non-mosaic form. The location of microduplication was confirmed with fluorescence *in situ* hybridization.

**Conclusion:**

The proband’s microduplication encompassed approximately 40 annotated genes, several of which have been associated with phenotypic characteristics of the proband. This is the first report of sSMC 17 including this particular chromosomal region in non-mosaic form.

## Background

Small supernumerary marker chromosomes (sSMC) are a morphologically heterogeneous group of structurally abnormal chromosomes that cannot be identified unambiguously by conventional cytogenetics. The definite origin of sSMC can only be diagnosed using molecular cytogenetic techniques [1]. sSMC frequency in newborns is 0.04% [2] but their clinical variability may range from normal to severely abnormal phenotype. The effects of sSMC may be attributed to their size, presence of euchromatic material and degree of mosaicism [3]. Uniparental disomy of sSMC’s sister chromosomes can also be identified [4]. The majority of sSMC cases arise *de novo* whilst 30% segregate within a family [3].

A *de novo* sSMC derived from a pericentric region of the q arm in chromosome 17 in a centric minute shape is described. It was identified postnatally and confirmed with fluorescence *in situ* hybridization (FISH). To our knowledge, this is the first report of this particular sSMC 17 in non-mosaic form.

## Case presentation

The 4 years old proband was born after 36 weeks of gestation to non-consanguineous healthy parents. The proband’s birth weight was 2600 g (10th-25th percentile), birth length 47 cm (25^th^-50th percentile), occipital-frontal circumference 32 cm (25th-50th percentile).

At the age of two months gastroesophageal reflux was diagnosed. She walked unaided at 18 months and started uttering her first words at the age of 3 years, when her pediatrician referred her for further development assessment. Her craniofacial features included round face, microstomia, small chin and down-slanting palpebral fissures. Lobules of both ears were small. Ultrasound examination of the heart and lung was unremarkable. Metabolic, endocrine and biochemical screening results were normal. Ophthalmologic examination showed myopia and anisometropia. Neurologic examination revealed mild general hypotonia with hypoplasia of orbicular oris muscle and levatorangulioris muscle. Her overall speech skills were delayed for at least 1.5 years. Her comprehension was limited to simple commands. Speech therapist was involved for further speech evaluation. She had a problem with concentration and visual-motoric coordination. Psychological examination revealed poor social interactions and severe hyperactivity. Other abnormal findings were supernumerary nipple on the left side, proximal placement of the thumbs and polycystic ovary. Dermatogliphes were normal; she had a small hypopigmented skin lesion on right thigh.

From neonatal period to the present age she didn't have significant health problems except recurrent otitis media. At first she attended kindergarten for children with developmental problems, but recently joined a regular kindergarten with additional speech and occupational therapy.

Once a year she is re-evaluated by the clinical geneticist. Her developmental abilities are constantly improving but are still delayed for her biological age. At the age of 4 she uses limited number of words in few-word sentences and understands simple commands. Her speech is sometimes still unclear and problems with hyperactivity persist. At the age of 4.2 years her developmental quotient (DQ) was calculated to be 0.68, representing significant development delay.

### Molecular and cytogenetic analysis

All cytogenetics and molecular-genetics analyses were performed with written and signed informed consent. Cytogenetic studies were carried out on peripheral blood lymphocytes from the patient and her parents. Cytogenetic analyses on metaphase-spread preparations were performed with standard procedures using GTG banding at a minimum 500 band resolution level according to the International System for Human Cytogenetic Nomenclature (ISCN 2013). Further molecular-cytogenetics analyses were undertaken using the FISH probe for chromosomal region 17q11.2 RP11-192H23 (BlueGnome, Illumina, San Diego, CA) on metaphase and interphase chromosome spreads. Chromosomes were counterstained with 4’,6-diamidino-2-phenylindole (DAPI) and images were captured using the CytoVysion Imaging System (Leica Biosystems. Wetzlar, DE).

Array comparative genomic hybridization (aCGH) analysis was performed on genomic DNA from a proband’s peripheral blood sample using a commercial oligonucleotide array (Agilent 60 K ISCA Oligo, Agilent Technologies, Santa Clara, CA) and sex-matched human reference DNA sample (Agilent Technologies, Santa Clara, CA). Data were analysed with the Cytogenomics 3.0 Software (Agilent Technologies, Santa Clara, CA). Peripheral blood lymphocytes from both parents were analysed by conventional karyotyping and additionally using specific 17q11.2 FISH probe RP11-192H23 to exclude the parental presence of sSMC or a balanced chromosomal rearrangement.

## Results

**Fig. 1:**
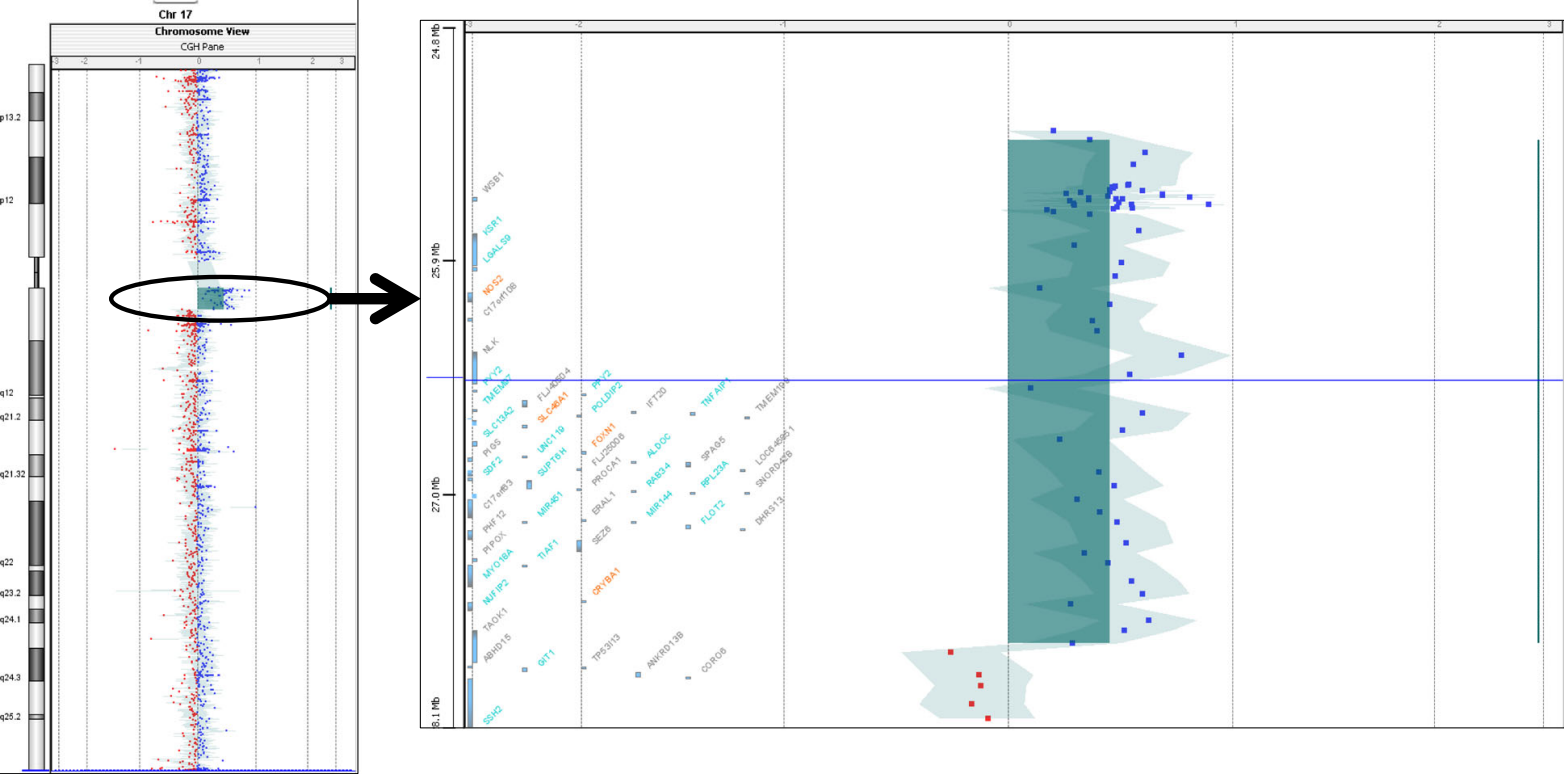
The result of aCGH indicating microduplication of chromosome 17 (arr[hg19]17q11.1q11.2(25,403,446-27,716,930)x3). Whole chromosome 17 and a close-up of duplicated region with annotated genes is visualized

**Fig. 2:**
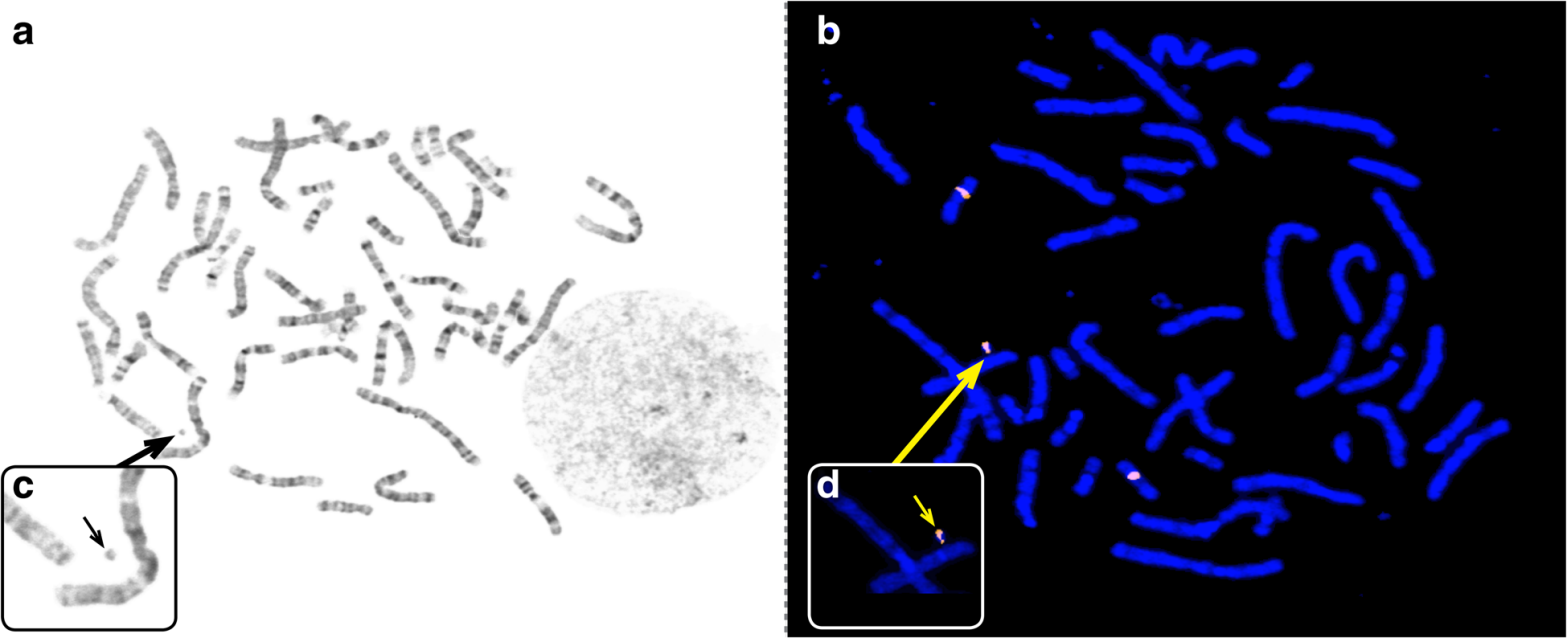
Results of high resolution G-banding (**a**) and FISH (**b**) with identified sSMC marked by arrows. The close-up of sSMC for corresponding metaphase is presented in both smaller frames (**c**, **d**). The orange FISH signals are the result of metaphase hybridization with the probe RP11-192H23 (BlueGnome, Illumina)

A postnatal aCGH showed a microduplication of 2.31 ± 0.06 Mb encompassing pericentric regions q11.1 and q11.2 derived from chromosome 17 (arr[hg19] 17q11.1q11.2(25,403,446-27,716,930)x3) (Fig. 1). Based on cytogenetic analysis of metaphases from peripheral blood we interpreted the karyotype as 47,XX,+der(17)(:p11.1- > q11.2:) in non-mosaic form. The location of microduplication was confirmed with molecular analysis using FISH probe RP11-192H23 (Fig. 2). Based on its shape, size and centromere we concluded that marker chromosome was a centric minute sSMC 17. Parental analysis demonstrated normal karyotypes and regular chromosomes 17 FISH patterns, consistent with a *de novo* origin of sSMC.

## Discussion

We found a *de novo* sSMC derived from chromosome region 17q11.1q11.2 using aCGH and FISH in a girl with developmental delay, speech delay, dysmorphic features and hyperactivity. According to the sSMC 17 database at Jena University Hospital, the proximal partial trisomy of q arm of chromosome 17 in general leads to developmental delay, dysmorphism, growth retardation, hypotonia, and visual impairment [5]. All these characteristics were observed in our proband. The duplicated region of cytoband 17q11.2 lies outside the potentially non dosage-sensitive pericentric region as confirmed by molecular mapping listed in sSMC database.

The formation of this *de novo* sSMC was probably due to a partial trisomy rescue and may be the result of viable postzygotic nondisjunction or anaphase lag event occurring during early embryogenesis [3, 6]. Advanced maternal age increases the incidence of meiotic non-disjunction events [7] and in our case the mother was 33 years old at the time of conception. There are numerous proposed models for centric minute sSMC formation but so far no pathways or enzymes involved in the processes of trisomy rescue mechanisms were discovered [3]. Although the majority of this life saving outcomes result in mosaic sSMC formation, the discrepancy in the level of mosaicism in different tissues of the same individual is well established. Trisomy rescue is also one of proposed mechanism for uniparental disomy (UPD). It has been estimated that *de novo* sSMC contribute to 4% of reported UPD cases [8]. However, UPD 17 is not regarded to be associated with any imprinting disorder. To date more than 3.300 cases have been reported in UPD database, yet there are no reports for coincidence of sSMC and mat/pat UPD 17 with clinical finding. Therefore, we interpreted this minute centric *de novo* sSMC 17 as resulting from a combination of meiosis non-disjunction event and incomplete trisomy rescue event in which the majority of additional chromosome 17 was lost. The remaining minute sSMC 17 harbours genomic gain which is entirely causative for the clinical phenotype.

The proband’s microduplication encompassed approximately 40 annotated OMIM genes, several of which were reported as potentially associated with proband’s phenotypic characteristics. Among clinically relevant genes within the duplicated region were *NOS2, TRAF4, SEZ6, SARM1* and *LGALS9*. Gene *NOS2* (OMIM 163730) encodes nitric oxidase synthase 2A that produces nitric oxide (NO), an important second messenger molecule involved in the regulation of cardiovascular, immune and neural system. NO has an array of functions in the central nervous system. In an association with cognitive function it has an important role in the induction and maintenance of synaptic plasticity [9]. Furthermore, the excess production of NO causes “nitrosative stress” and consequent neurotoxicity. NO also acts at the level of transcription and translation and regulates cell survival and proliferation in diverse cell types, including neuron cells [10]. So, it affects neurogenesis, where the continuous formation and pruning of neuron synapses are the main processes. Second gene *TRAF4* (OMIM 602464) encodes TNF receptor-associated factor 4, which is required during embryogenesis for the formation of the trachea, the axial skeleton and the closure of neural tube. In addition, *TRAF4* plays a role in proper myelination therefore its role in ADHD, intellectual disability and movement disorder has been previously suggested [11]. Other genes within the duplicated region were also of interest for possible genotype-phenotype correlations. Gene *SEZ6* (OMIM 616666) is predicted to be involved in neuronal maturation and plasticity, based on animal studies [12]. *Mary et al.* reported that *SEZ6* expression correlates with the most active periods of cortical neurogenesis and neuronal maturation [13]. Gene *SARM1* (OMIM 607732) activity is required after axon injury to induce axon degeneration in mice [14], while *LGALS9* gene (OMIM 601879) encodes different lectins. Specific interactions between carbohydrate moieties and their putative binding proteins (i.e. lectins) play a critical role in various developmental, physiologic, and pathologic processes. Based on the description and the mechanism of genes included in this sSMC, we suggest that duplication of *NOS2* and *TRAF4* genes play a crucial role in our patient. Altered NO production in brain may affect neurotoxicity and eventually influence neurogenesis. It results in an inappropriate development and working of the whole central nervous system, which could be a reason for the proband’s developmental delay. It has been shown that *TRAF4* is attractive gene for movement disorders [11], so we can suggest that duplication of *TRAF4* is probably involved in our patient’s hyperactivity. This observation remains speculative and needs to be confirmed in other similar cases or functional studies. Currently, there are no published reports for *SEZ6, SARM1* and *LGALS9* involvement into development of developmental delay or dysmorphism and their role in the etiology of presented case is unclear. The comprehensive study of all annotated genes in the duplicated region was performed but no candidate genes for supernumerary nipple or polycystic ovary was identified. However, the function of many genes in the duplicated region is currently not known.

To date, not a single reported case with the sSMC 17 encompassing this particular coordinates of pericentric region of q11.1 and q11.2 was identical to ours. In Table 1 we present overlapping cases from the literature. The presence of sSMC 17 encompassing only the pericentric regions appears to be associated predominantly with developmental delay and in some cases with intellectual disability, meanwhile patients with sSMC 17 of larger parts of either the short or the long arm of chromosome 17 have additional dysmorphic features [11]. The duplicated genomic region of the present case is the most comparable to that of *Chen et al.*[15], although our proband’s heart examination did not reveal any abnormalities. Both cases manifested with developmental delay and speech delay, despite the fact that sSMC 17 in the *Chen et al.* case was in a mosaic form. *Cornelius et al.* described another similar case [11] and suggested that the duplication of *NOS2* plays a role in the ADHD symptoms of their patient. *Cornelius et al.* also suggested that the duplication of *TRAF4* plays a role in ADHD, intellectual disability and movement disorder. Although the present proband does not fulfill all the criteria of ADHD, she has a severe form of hyperactivity.

**Table 1:** Clinical cases of sSMC 17

Author	Final result of sSMC 17	Clinical symptoms	Ref.
*Chen et al.*	17q11.1q11.2 (25,372,965-27,725,134) in mosaic form; prenatal diagnosis	ventricular septal defect, developmental delay, speech delay with language problems at neonatal follows-up	[15]
*Cornelius* *et al.*	17p11.2q11.2 (21,200,000-27,500,000) in mosaic form	22-year-old male with Gilles de la Tourette syndrome, attention deficit hyperactivity disorder (ADHD), intellectual disability and seizures	[11]
*Vetro et al.*	sSMC(17) involving three duplications separated by two single copy regions with a size of about 2.1 Mb and 615 kb (NCBI 36): 17p11.2 (16,892,427-19,888,467) (2,9 Mb),17q11.1 (22,427,573-23,163,556) (319 kb) and 17q11.2 (23,848,894-25,676,268) (1,8 Mb)in mosaic form	severe global developmental delay, speech delay, hypotonia, microcephaly and mild dysmorphic features	[16]
*Kozma et al.*	r(17)(::p11.1 → q21::) in mosaic form; no coordinates given	38-year-old male with developmental delay, profound mental retardation, kyphoscoliosis, bilateral cataracts, severe calcaneovalgus deformity of the feet, dysmorphic facies, mitral valve prolapse with regurgitation and severe respiratory insufficiency. He never developed any speech.	[17]
*Capovia P.* *et al.*	min(17)(:p11.1/q11.2:) in size of 10 Mb; in mosaic form	2-year-old male with minor facial dysmorphic features, developmental delay (especially speech delay), short stature and Potocki-Lupski syndrome	[18]
*Jason Anderson*	min (17) (23,086,100-32,754,790 MB)	developmental delay in newborn	[5]
*Manolakos* *et al.*	min(17) of three duplications: (p11.2 → p11.2; 16.9-19.89) (p11.1 → q11.1; 22.42-23.16) (q11.2 → q11.2; 23.84-25.67) in non-mosaic form	significantly delayed developmental milestones (sitting unsupported at 12 months, walking unaided at 22 months, started uttering first words at 2.5 years), mild dysmorphic facial and body features (microcephaly, narrow palpebral fissures, small eyes, high-arched palate, low set ears, short hands and fingers and clinodactyly of the 5th finger), severe hypotonia, microcephaly, IQ <46 (at 17 months)	[19]
*Neill NJ* *et al.*	mar(17) (:p11.1 → q11.2:) in mosaic form	abnormal	[20]

## Conclusion

We report a *de novo* sSMC derived from 17q11.1q11.2 in association with developmental delay, speech delay and mild dysmorphism. This unique clinical case was compared to published overlapping cases. We conclude that the identification of the origin, the size and the composition of sSMC using aCGH and FISH are essential for precise genotype-phenotype correlation. Furthermore, thorough molecular-cytogenetic characterization of sSMC is needed for the prediction of clinical prognosis and genetic counseling.

## Abbreviations

*aCGH*: Array comparative genomic hybridization
*sSMC*: Small supernumerary marker chromosome
*FISH*: Fluorescent *in situ* hybridization
*GTG*: Giemsa-Tripsin-Gbanding
